# Fronto-striatal organization: Defining functional and microstructural substrates of behavioural flexibility

**DOI:** 10.1016/j.cortex.2015.11.004

**Published:** 2016-01

**Authors:** Laurel S. Morris, Prantik Kundu, Nicholas Dowell, Daisy J. Mechelmans, Pauline Favre, Michael A. Irvine, Trevor W. Robbins, Nathaniel Daw, Edward T. Bullmore, Neil A. Harrison, Valerie Voon

**Affiliations:** a Department of Psychology, University of Cambridge, Cambridge, United Kingdom; b Behavioural and Clinical Neuroscience Institute, University of Cambridge, Cambridge, United Kingdom; c Department of Psychiatry, University of Cambridge, Addenbrooke's Hospital, Cambridge, United Kingdom; d Section on Functional Imaging Methods, National Institute of Mental Health, Bethesda, MD, USA; e Department of Psychiatry, Brighton and Sussex Medical School, Brighton, United Kingdom; f Laboratory of Psychology and Neurocognition, University Grenoble Alpes, Grenoble, France; g Center for Neural Science and Department of Psychology, New York University, New York, NY, USA; h Cambridgeshire and Peterborough NHS Foundation Trust, Cambridge, United Kingdom; i NIHR Cambridge Biomedical Research Centre, Cambridge, United Kingdom

**Keywords:** Fronto-striatal loops, Goal-directed, Habit, Microstructure, Neurite density

## Abstract

Discrete yet overlapping frontal-striatal circuits mediate broadly dissociable cognitive and behavioural processes. Using a recently developed multi-echo resting-state functional MRI (magnetic resonance imaging) sequence with greatly enhanced signal compared to noise ratios, we map frontal cortical functional projections to the striatum and striatal projections through the direct and indirect basal ganglia circuit. We demonstrate distinct limbic (ventromedial prefrontal regions, ventral striatum – VS, ventral tegmental area – VTA), motor (supplementary motor areas – SMAs, putamen, substantia nigra) and cognitive (lateral prefrontal and caudate) functional connectivity. We confirm the functional nature of the cortico-striatal connections, demonstrating correlates of well-established goal-directed behaviour (involving medial orbitofrontal cortex – mOFC and VS), probabilistic reversal learning (lateral orbitofrontal cortex – lOFC and VS) and attentional shifting (dorsolateral prefrontal cortex – dlPFC and VS) while assessing habitual model-free (SMA and putamen) behaviours on an exploratory basis. We further use neurite orientation dispersion and density imaging (NODDI) to show that more goal-directed model-based learning (MB_c_) is also associated with higher mOFC neurite density and habitual model-free learning (MF_c_) implicates neurite complexity in the putamen. This data highlights similarities between a computational account of MF_c_ and conventional measures of habit learning. We highlight the intrinsic functional and structural architecture of parallel systems of behavioural control.

## 1 Introduction

Mapping the functional organization of cortico-basal ganglia-thalamo-cortical (CBGTC) circuit connectivity is crucial as it aids our understanding of behaviour, motor control and the emergence of neuropsychiatric disorders. Fronto-striatal circuitry can be broadly divided into motor, limbic and cognitive projections (Groenewegen et al., 1999, Haber, 2003). In humans, resting state connectivity studies have used several methods to analyse fronto-striatal coupling including defining cortical projections from 6 striatal seeds (Di Martino et al., 2008) and striatal mapping using clustering algorithms of the entire cerebral cortex (Choi et al., 2012, Jung et al., 2014). Here, we extend these studies by developing connectivity maps based on carefully defined prefrontal seed regions (based on function) and following striatal functional projections through the basal ganglia and thalamus. We use a novel multi-echo planar imaging sequence and independent components analysis (ME-ICA) that greatly enhances signal-to-noise ratios compared to single-echo sequences thus allowing higher spatial resolution of subcortical structures (Kundu, Inati, Evans, Luh, & Bandettini, 2012).

We further examine the functional relevance of these connections by assessing the behavioural correlates of fronto-striatal connectivity, focussing on goal-directed behaviours and attentional set shifting, with an exploratory focus on cognitive-behavioural flexibility in the form of reversal learning for both reward and loss and separately, habitual behaviour. The capacity to flexibly adapt behaviour is crucial to negotiating the vicissitudes of daily life. Behaviour is believed to be a product of parallel decisional systems. On the one hand, flexible goal-directed behaviour is guided by the assessment of a model of environmental contingencies and remains sensitive to outcome value, whereas habitual behaviour entails decisions that are made based on previously reinforced actions (Daw, Gershman, Seymour, Dayan, & Dolan, 2011). Although most of us seem to effortlessly blend or alternate between the two systems, several pathological disorders have been associated with their imbalance (Everitt and Robbins, 2005, Gillan et al., 2011, Sjoerds et al., 2013, Voon et al., 2014, de Wit et al., 2012). Recent computational theories describe two distinct forms of learning known as model-based and model-free reinforcement learning. These provide a computational framework which is hypothesized to underlie goal-directed and habitual behaviours, respectively (Dayan & Niv, 2008). We focus on mapping the intrinsic functional connections, as well as neural microstructure features, associated with model-based and MF_c_ in healthy volunteers.

Goal-directed behaviour has been explored using lesion studies in rodents and imaging studies in humans, particularly implicating the ventromedial prefrontal and orbitofrontal cortices (Balleine and O'Doherty, 2010, Yin et al., 2005). In contrast, to the ventromedial prefrontal cortex (vmPFC), which encodes action-outcome contingencies and action values to guide behaviour (Glascher et al., 2009, Tanaka et al., 2008, Wunderlich et al., 2012, Yin et al., 2005), the orbitofrontal cortex (OFC) is involved in the computation and updating of outcome value in the context of changing internal motivational states or feedback (J. S. Morris and Dolan, 2001, O'doherty et al., 2001, Valentin et al., 2007). The OFC, ventral striatum (VS) and also amygdala respond not only to primary (food, drugs), but also to secondary rewards (money) (Haber and Knutson, 2010, Kringelbach and Rolls, 2003, Shin et al., 2013). Medial-lateral divisions within the OFC are apparent, with medial regions involved with reward and value monitoring and lateral regions becoming recruited when an action previously associated with reward must be suppressed (important for reversal learning) (Elliott et al., 2000, O'doherty et al., 2001). Anticipation and response to negative outcomes has been associated with the anterior insula (Seymour, Daw, Dayan, Singer, & Dolan, 2007), with activity within this region also predicting behavioural avoidance to losses (Kuhnen and Knutson, 2005, Samanez-Larkin et al., 2008). The nucleus accumbens or VS, receives extensive anatomical connections from OFC(Groenewegen et al., 1999, Stefanacci and Amaral, 2002) and encodes anticipation and receipt of reward, tracking prediction error and linking motivationally-relevant reward properties with instrumental performance and response vigour (Corbit et al., 2001, Pessiglione et al., 2006, Schultz et al., 1997, Talmi et al., 2008). While a previous study implicated anatomical connectivity of the caudate nucleus in flexible goal-directed behaviour assessed via ‘slips of action’ (de Wit et al., 2012), ventral striatal activity has been linked to model-based valuation, as well as model-free reward prediction error (Daw et al., 2011). Furthermore, model-based behaviour has been associated with higher grey matter volume, particularly in the medial orbitofrontal cortex (mOFC) (Voon et al., 2014). Thus, the medial OFC and VS have been implicated in model-basedness and may act via linking outcome valuation-updating and reward-related motivation, vital for such behavioural adaptations.

During the course of affective learning, a gradient shift of information processing from ventromedial to dorsolateral striatum (equivalent to human posterior putamen) is believed to occur (Everitt & Robbins, 2005). Correspondingly, actions progress from goal-directed to habitual, becoming repetitive, reliant on previously reinforced actions that are divorced from an apparent goal and persist despite outcome devaluation (Dickinson, 1985). The neural correlates of habit have predominantly focused on over-training and testing in devaluation in both rodent and human. Lesions of the dorsolateral striatum in rodents impair the ability to form and maintain habitual responding (Yin, Knowlton, & Balleine, 2004). Similarly in humans, progressive instrumental conditioning (Tricomi, Balleine, & O'Doherty, 2009) and habitual ‘slips of action’ (de Wit et al., 2012) following over-training are associated with a transition towards greater engagement of posterior putamen and its connectivity with premotor cortical regions (de Wit et al., 2012). However, the neighbouring supplementary motor complex or SMC (comprising supplementary motor area – SMA and pre-SMA) is the seat of the cortical-subcortical motor circuit which primarily projects to putamen or dorsolateral striatum and is responsible for learning of stimulus-response contingencies (Chen and Wise, 1995, Nachev et al., 2008), associated with the development of inflexible and habitual behaviours towards drugs of abuse (Alexander et al., 1986, Everitt and Robbins, 2005, Kreitzer and Malenka, 2008). Thus, in terms of model-free behaviour, we expect a putaminal and SMA network to be implicated.

We employ a two-step sequential learning task used previously to show simultaneous engagement of model-based and MF_c_ in healthy volunteers (Daw et al., 2011) and their imbalance in pathological states associated with compulsivity (Voon et al., 2014). The task involves decision preferences that evolve on a trial-by-trial basis differing depending on which of the two sorts of learning predominates. Subjects choose between stimulus-pairs at Stage 1, which leads, according to a fixed probabilistic schedule (*p* = .70), to one of two stimulus-pairs at Stage 2. Choice of a stimulus at Stage 2 is associated with a gradually shifting probability (*p* = .25–.75) of reward. For MF_c_, a reward prediction error reinforces the associated Stage 1 action. In contrast, for model-based learning (MB_c_), a prospective model of stage transitions is built incorporating updated Stage 2 values to drive Stage 1 choices. While the relative contribution of model-based and model-free can be captured by a single parameter, *w*, (model-based, *w* = 1; model-free, *w* = 0) the discrete characteristics of these separate forms of predictions and learning allow their computational and behavioural influences to be teased apart. In both functional task-based and anatomical imaging studies, a computation account of MB_c_ has been associated with medial OFC (Daw et al., 2011, Glascher et al., 2010, Lucantonio et al., 2012, Voon et al., 2014). Prediction error activity for both model-based and MF_c_ converges on the VS (Daw et al., 2011, Glascher et al., 2010, Lucantonio et al., 2012, Voon et al., 2014).

In Study 1, we aimed to map CBGTC functional connections by examining fronto-striatal and striatal – basal ganglia and thalamic connections. In Study 2, we build upon this by assessing the functional neural correlates of well-established measures of behavioural flexibility, namely model-based behaviour or *w* in healthy volunteers and on an exploratory basis we also examine the correlates of model-free behaviour. To further the functional characterization of frontal-striatal connections, we additionally assess probabilistic reversal learning and attentional set shifting as both processes have evidenced dissociability of frontal cortical involvement; lateral OFC is required for the implementation of reversal (Hampshire, Chaudhry, Owen, & Roberts, 2012) whereas attentional shifting implicates dorsolateral prefrontal cortex (dlPFC) (Dias et al., 1996, Hampshire et al., 2012, Manes et al., 2002). Thus, we hypothesize associations with connectivity of the lateral OFC with VS, and dlPFC with striatum, respectively.

In Study 3, we acquired neurite orientation dispersion and density imaging (NODDI) data from a separate cohort of healthy volunteers to further examine the microstructural correlates of *w*. NODDI is a recently established technique that characterizes features of the underlying tissue microstructure with better specificity than a typical diffusion tensor imaging (DTI) approach. For example, NODDI computes parameters such as neurite density and orientation dispersion, while DTI combines this information into a single fractional anisotropy (FA) value. The current approach has a more direct relationship with axonal and dendritic orientation distribution (Jespersen, Leigland, Cornea, & Kroenke, 2012), as well as neurite density and dendritic architecture (Jespersen et al., 2010). Increasing dendritic complexity and density is associated with hierarchical computational capacity of cortical structures (Jacobs et al., 2001). In white matter, orientation dispersion captures the bending and fanning of fibres, important for determining anatomical connectivity (Kaden, Knosche, & Anwander, 2007), while in grey matter it captures sprawling dendritic processes, providing a more accurate measure of grey matter complexity (Zhang, Schneider, Wheeler-Kingshott, & Alexander, 2012). In contrast to DTI (which assumes Gaussian diffusion within a single compartment), NODDI employs a three-compartment model (Panagiotaki et al., 2012) that represents three distinct microstructural environments within tissue: (1) the intracellular space, where water diffusion is restricted by dendritic or axonal membranes and follows a non-Gaussian pattern of displacement, which is modelled by zero-radius sticks with orientation dispersion index (ODI) determined by the Watson distribution (Mardia and Jupp, 1990); (2) extracellular space, where water diffusion is hindered by the presence of glial and cell body (soma) membranes and has a Gaussian anisotropic displacement and is modelled by orientation dispersed cylinders (Zhang, Hubbard, Parker, & Alexander, 2011); and (3) the cerebrospinal fluid (CSF) space where water diffusion is unhindered and isotropic (Zhang et al., 2012). In line with the discussed literature, we primarily hypothesize that MB_c_ is associated with higher microstructural density and complexity of the medial OFC and VS. On an exploratory basis, we also assess MF_c_ hypothesizing greater microstructural density and complexity of the SMA and putamen.

## 2 Materials and methods

### 2.1 Participants

We recruited healthy volunteers from community and University-based advertisements in the East Anglia region, United Kingdom. Participants were excluded if they had current major depression or other major psychiatric disorder, substance addiction or major medical illness or were taking psychotropic medications. Psychiatric disorders were screened with the Mini International Neuropsychiatric Interview (Sheehan et al., 1998). The National Adult Reading Test was used to assess IQ (intelligence quotient). Participants were compensated for their time and paid an additional amount depending on their performance. Written informed consent was obtained and the study was approved by the University of Cambridge Research Ethics Committee. Participants completed the Beck Depression Inventory to assess depressive symptoms. For Study 1, 66 healthy volunteers [33 male; mean age 40 (SD 13) years old; Beck Depression Inventory 8.6 (SD 8.4); verbal IQ 114.3 (SD 9.5)] underwent a resting-state functional MRI scan and for Study 2, the same HV (healthy volunteer) completed the three behavioural tasks outside of the scanner. Thirty-seven healthy volunteers [17 male; mean age, 36.6 (SD 14.1); verbal IQ, 113.0 (SD 11.7)] completed the probabilistic reversal learning task. Participants completed the resting-state functional MRI scan and behavioural testing no more than 7 days apart. For study 3, a separate cohort of healthy volunteers (38 healthy volunteers: 24 female; age 23.5, 4 SD; 115 verbal IQ, 6.7 SD) underwent a NODDI scan and completed the model-based and model-free task outside the scanner.

### 2.2 Model-free model-based task

To assess goal-directed (model-based) and habitual (model-free) learning strategies, we used a two-step choice task (Daw et al., 2011) (Fig. 6A). Subjects underwent extensive computer-based training in the instructions phase. At stage 1, participants chose between a stimulus pair, leading with fixed probability to one of two states at stage 2. At the second stage, participants chose between two stimuli, each associated with differing probabilities of reward. The probability shifted gradually over the course of the task. Each stage lasted 2 sec, the transition 1.5 sec and the outcome 2 sec. Using a computational algorithm, habit learning was modelled using a model-free reinforcement learning algorithm and goal-directed learning modelled using an algorithm taking into account the model transitions. A weighting factor can be calculated for each individual, describing the relative contribution of either habitual (model-free, MF, *w* = 0) or goal-directed (model-based, MB, *w* = 1) decision-making. The primary outcome measure was the relationship between w and dendrite orientation, neurite density and functional connectivity. For *a priori* defined regions, *p* < .05 was considered significant. In secondary analyses, we also assessed the computational habitual and goal-directed elements which were further separable by computing MFc = *β*1*(1 − *w*) and MBc = *β*1*w respectively and behavioural model-based (MB_b_) and model-free (MF_b_) scores (see Supplementary materials for further details). The task was programmed with Matlab 2011a.

### 2.3 Extra-dimensional (ED) set-shifting

The Intra/Extra-dimensional set shifting task (Cambridge Neuropsychological Test Battery) is a choice discrimination task testing rule acquisition and reversal (Downes et al., 1989) (Fig. 7A). After six consecutive correct responses, the rules are changed. For the ED set shift a previously irrelevant stimulus dimension becomes relevant and participants must make a conceptual attentional shift, requiring cognitive flexibility (see Supplementary materials for further details). The primary outcome measure was the number of ED errors.

### 2.4 Probabilistic reversal task

The probabilistic reversal learning task consisted of an acquisition and reversal phase, each with three conditions of varying reward, neutral or loss outcomes. In the acquisition phase, subjects chose from 3 stimulus-pairs associated with probabilistic outcomes (described further in Supplemental materials). Following 30 trials of each condition for acquisition, the contingencies for each stimulus-pair switched and were thereafter followed by 30 trials per condition in the reversal phase. The stimulus phase (2.5 sec) was followed by an outcome phase (1 sec) with the feedback; “You WON!!” with an image of a £2 or £1 coin or “You LOST!!” with an image of a red cross over the money. The trial was followed by a variable inter-trial interval of a mean of .75 sec varying between .5 and 1 sec. Subjects were instructed to choose between pairs of symbols and that one symbol within each pair was more likely to win or not lose money and that at some point the relationship between the symbols and the likelihood of winning and not losing money might change. Subjects were told to make as much money as possible of which a proportion would be paid to them at the end of the study. The primary outcome measure was the number of trials to criterion of 4 correct sequential choices. The task was coded in E-prime Version 2.

### 2.5 Resting-state functional MRI data acquisition and analysis

In order to examine the fronto-striatal connectivity organization and the underlying behavioural correlates, we analysed blood-oxygenation level dependent (BOLD) fMRI data during rest in all participants. To enhance signal-to-noise ratio, we employed a novel ME-ICA in which BOLD signals were identified as independent components having linear TE-dependent signal change and non-BOLD signals were identified as TE-independent components (Kundu et al., 2012).

Data was acquired with a Siemens 3T Tim Trio scanner using a 32-channel head coil at the Wolfson Brain Imaging Centre at the University of Cambridge. Anatomical images were acquired using a T1-weighted magnetization prepared rapid gradient echo (MPRAGE) sequence (176 × 240 FOV – field of view; 1-mm in-plane resolution; inversion time (TI), 1100 msec). All participants underwent a resting-state fMRI scan of 10 min. Functional images were acquired with a multi-echo echo planar imaging sequence with online reconstruction (repetition time – TR, 2.47 sec; flip angle, 78°; matrix size 64 × 64; in-plane resolution, 3.75 mm; FOV, 240 mm; 32 oblique slices, alternating slice acquisition slice thickness 3.75 mm with 10% gap; iPAT factor, 3; bandwidth – BW = 1,698 Hz/pixel; TE = 12, 28, 44 and 60 msec).

Multi-echo independent component analysis (ME-ICA v2.5 beta6; http://afni.nimh.nih.gov) was used for analysis and denoising of the multi-echo resting-state fMRI data. ME-ICA initially decomposes multi-echo fMRI data into independent components using FastICA. Then, independent components are categorized as BOLD or non-BOLD based on their weightings measured by Kappa and Rho values, respectively. BOLD signal has percent signal changes that are linearly dependent on echo time (TE), a characteristic of the T2* decay. TE dependence of BOLD signal is measured using the pseudo-*F*-statistic, Kappa, with components that scale strongly with TE having high Kappa scores (Kundu et al., 2012). Non-BOLD components are identified by TE independence measured by the pseudo-*F*-statistic, Rho. By removing non-BOLD components (by projection), data are denoised for motion, physiological and scanner artefacts in a robust manner based on physical principles (Kundu et al., 2013). Each individual's denoised echo planar images (EPI) are coregistered to each individual's MPRAGE and normalized to the Montreal Neurological Institute template.

Functional connectivity analysis was performed using a regions of interest (ROI)-driven approach with CONN-fMRI Functional Connectivity toolbox (Whitfield-Gabrieli & Nieto-Castanon, 2012) for Statistical Parametric Mapping SPM8 (http://www.fil.ion.ucl.ac.uk/spm/software/spm8/). Spatial smoothing was conducted with a Gaussian kernel (full width half maximum = 6 mm). The time course for each voxel was temporally band-pass filtered (.008 < *f* < .09 Hz). Each individual's anatomical scan was segmented into grey matter, white matter and CSF. Significant principal components of the signals from white matter and CSF were removed.

We used strictly and carefully defined ROI's, for example: the medial and lateral OFC were distinguished by the crown of the gyrus rectus (Cox et al., 2014); the vmPFC was defined by the posterior border of the anterior prefrontal cortex (antrPFC) (Ongur, Ferry, & Price, 2003), the cingulate cortex, the genu of the corpus callosum and the superior boundary of the medial OFC; the dlPFC was based on Brodmann areas 46 and 9; the border between pre-SMA and SMA was defined as a vertical line through the anterior commissure; the subgenual cingulate (sgACC) was based on Brodmann Area 25; the dorsal anterior cingulate cortex (dACC) was restricted to the tip of the genu of the corpus callosum (Cox et al., 2014, Desikan et al., 2006) and posterior end of the genu of the corpus callosum (Desikan et al., 2006); the inferior frontal cortex (IFC) was bordered by the inferior frontal sulcus, the precentral gyrus (Cox et al., 2014, Desikan et al., 2006) and the rostral extent of the inferior frontal sulcus (Desikan et al., 2006); the antrPFC was based on Brodmann area 10 and manually restricted at the boundary of the anterior coronal place where the three frontal gyri are present (Ongur et al., 2003, Ongur and Price, 2000, Ramnani and Owen, 2004), and by the dorsal extent of area 10p described by (Ongur et al., 2003); the dorsomedial prefrontal cortex (dmPFC) used the dorsal boundary of the anterior PFC and the lateral boundaries described for the vmPFC. Further extensive details for these definitions are detailed in Supplementary materials.

**Fig. 1:**
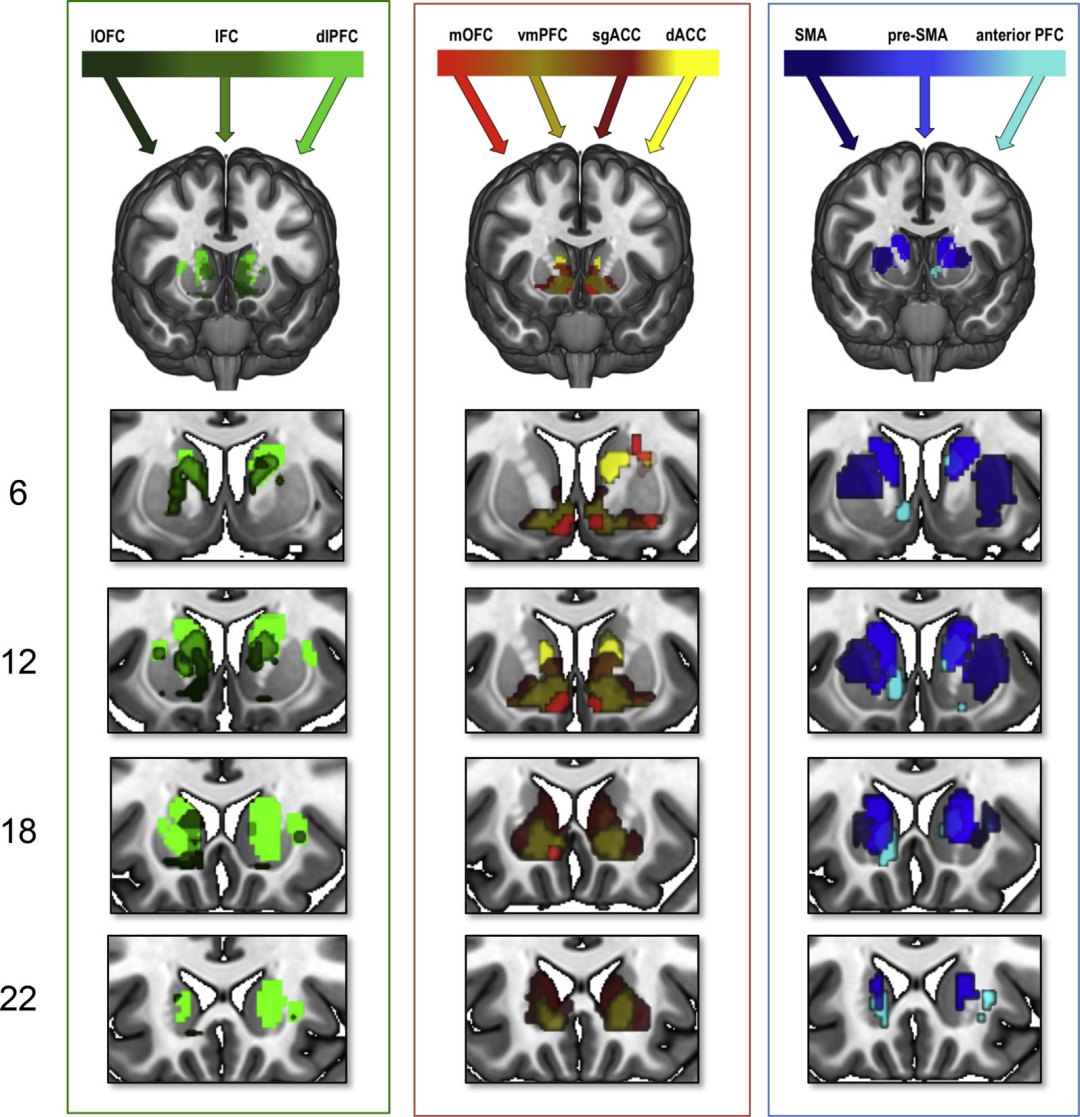
Intrinsic fronto-striatal connectivity. Prefrontal seeds are illustrated (top) with striatal connectivity colour-coded to the prefrontal seeds. Several additional enlarged slices are included below each fronto-striatal connectivity map by coronal slice number along the y direction (left). The blood-oxygen-level dependent (BOLD) overlays are illustrated with a striatal mask at family wise error corrected *p* < .005 (medial orbitofrontal cortex shown at *p* < .05) for illustration purposes. Abbreviations: IFC: inferior frontal cortex, dlPFC: dorsolateral prefrontal cortex, lOFC: lateral orbitofrontal cortex, mOFC: medial orbitofrontal cortex, vmPFC: ventromedial prefrontal cortex, sgACC: subgenual cingulate, dACC: dorsal cingulate, SMA: supplementary motor area, pre-SMA: pre-supplementary motor area, anterior PFC: anterior prefrontal cortex.

**Fig. 2:**
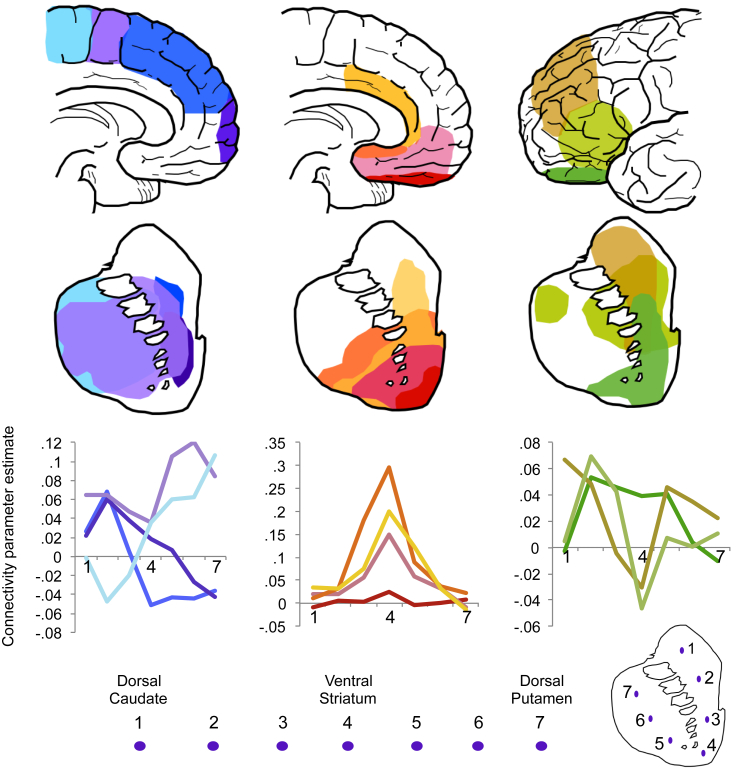
Schematic depiction of intrinsic fronto-striatal connectivity. Top: schematic illustration of cortical seeds with regions of striatal connectivity for corresponding colour-coded cortical seeds below. For the purposes of illustration and comparison, the maps were reproduced from images using a striatal mask set at a threshold of FWE corrected *p* < .005 (medial orbitofrontal cortex to ventral striatum (red) was based on a threshold of FWE corrected *p* < .05 and pre-supplementary motor area connectivity with putamen/caudate (light purple) was based on a threshold of FWE corrected *p* < .0001). Bottom: Parameter estimates of connectivity for each cortical seed are plotted in the same colour-coded system for 7 points along the striatum from dorsal caudate (point 1) to ventral striatum (point 4) to dorsal putamen (point 7).

We examined intrinsic fronto-striatal connectivity by computing the beta maps of ROI-to-voxel (whole brain) analysis for each cortical seed region and restricted the observation of functional connectivity to the whole striatum, controlling for age. Exploratory analyses of gradient patterns through the striatum were performed for cortical regions of interest with heterogeneous striatal connectivity, namely dlPFC, pre-SMA and SMA. First, parameter estimates of connectivity for each frontal cortical seed with the striatum were computed at 7 points along coronal slice 12 (Fig. 1) of the right striatum. The coordinates were chosen to be 5 mm from the top of the caudate (point 1, xyz = 15, 12, 16) and putamen (point 7, xyz = 25, 12, 4), with approximately 8 mm between each of the 7 points. Thus, connectivity parameters were extracted from the following discrete striatal points: 1. Dorsal caudate; 2. Mid caudate; 3. Ventral caudate; 4. VS (xyz = 11, 12, −10); 5. Ventral putamen; 6. Mid putamen; 7. Dorsal putamen. Connectivity estimates for mid caudate (point 2), VS (point 4) and mid putamen (point 6) for named cortical regions of interest were entered into one-way ANOVA for statistical comparisons. See Fig. 2 for a schematic of their positioning. A similar approach was taken to examine an anterior – posterior gradient of cortical connectivity within putamen. Five points were chosen 6 mm apart along the putamen in axial plane 0 (Fig. 4), with the most anterior position at xyz = 26, 18, 0 and posterior at xyz = 32, −6, 0. Connectivity estimates for anterior (point 1), mid (point 3) and posterior (point 5) putamen were entered into one-way ANOVA to compare between cortical connectivity strengths. See Supplemental material for further details.

For basal ganglia and thalamic mapping, the 3 major striatal subregions were used as seeds and functional connectivity was restricted to each basal ganglia subregion and thalamus. For this, we used small volume corrected family wise error (SVC FWE).

For examination of behavioural functional correlates, given our strict *a priori* hypotheses, we computed ROI-to-ROI functional connectivity on an individual level. ROI-to-ROI correlation coefficients for the discussed region pairs were obtained by computing Pearson's correlation coefficients between BOLD time courses of ROI pairs. These correlation coefficients were then correlated with the behavioural measures described with age as a covariate of no interest. For our *a priori* hypotheses, *p* < .05 for the ROI-to-ROI analysis was considered significant. For the probabilistic reversal learning task correlates, we used specifically defined seed regions to compute ROI-to-voxel connectivity maps, which were entered into second level correlation analysis to assess associations with the behavioural measure. Due to the exploratory nature of this analysis and the implication of several cortical and subcortical regions in reversal learning and loss process, whole brain voxel-wise correlations were performed and cluster extent threshold correction was used. The cluster extent correction was calculated at 15 voxels at *p* < .001 whole brain uncorrected, which corrects for multiple comparisons at *p* < .05 assuming an individual-voxel Type I error of *p* = .01 (Slotnick, Moo, Segal, & Hart, 2003).

### 2.6 NODDI data acquisition and analysis

NODDI data optimized for the scanner was acquired from 38 healthy volunteers. Data was acquired with a Siemens 3T Tim Trio scanner using a 32-channel head coil at the Wolfson Brain Imaging Centre at the University of Cambridge with the following parameters: TE = 128 msec; TR = 11,300 msec; planar FOV = 192 mm × 192 mm; 96 matrix with 2 mm voxel and 2 mm slice thickness. There were 63 slices (b-values: 2850 and 700 sec/mm^2^ with 65 and 33 directions, respectively). A NODDI microstructural model was computed and fitted to the data using the NODDI toolbox for Matlab (Zhang et al., 2012, http://www.nitrc.org/projects/noddi_toolbox). The resulting parameter maps were normalized into MNI space using ANTS software (http://stnava.github.io/ANTs/) and masked before smoothing. Statistical Parametric Mapping SPM8 (http://www.fil.ion.ucl.ac.uk/spm/software/spm8/) was used for spatial smoothing (with a Gaussian kernel of full width half maximum = 6 mm) and second-level analysis. ODI maps and neurite density maps were entered into second level factorial analysis designs to assess group-level statistics. With examinations of specific regions of interest, SVC FWE *p* < .05 for the primary outcome was considered significant.

## 3 Results

We characterized CBGTC circuits in the form of frontal – striatal and striatal – basal ganglia – thalamic functional connections. We further confirmed the functional relevance of frontal-striatal connections by examining the correlates of well-established behaviours including goal-directed model-basedness and attentional shifting. On an exploratory basis we also examined probabilistic reversal learning for reward and loss, and habitual model-free behavioural correlates.

### 3.1 Study 1: intrinsic CBGTC connectivity

**Fig. 3:**
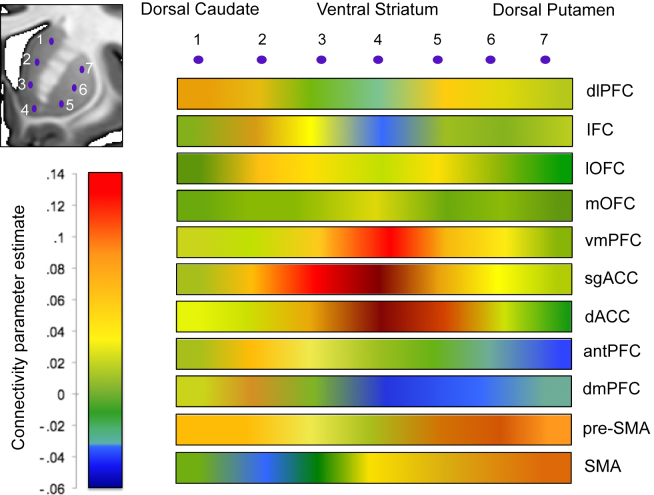
Fronto-striatal connectivity patterns as heat maps. Parameter estimates of connectivity for each cortical seed are illustrated as heat maps for 7 points along the right striatum from dorsal caudate (point 1) to ventral striatum (point 4) to dorsal putamen (point 7). Abbreviations: IFC: inferior frontal cortex, dlPFC: dorsolateral prefrontal cortex, lOFC: lateral orbitofrontal cortex, mOFC: medial orbitofrontal cortex, vmPFC: ventromedial prefrontal cortex, sgACC: subgenual cingulate, dACC: dorsal cingulate, SMA: supplementary motor area, pre-SMA: pre-supplementary motor area, antPFC: anterior prefrontal cortex; dmPFC, dorsomedial prefrontal cortex.

**Fig. 4:**
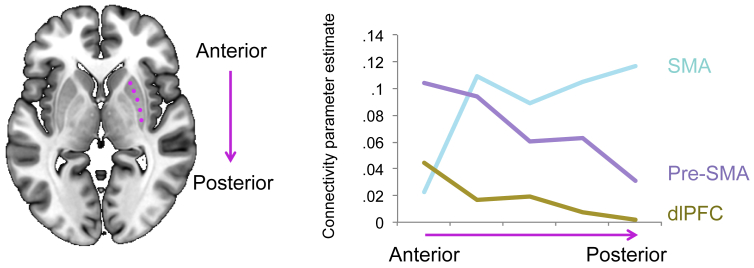
An anterior – posterior gradient of connectivity along the putamen. Parameter estimates of connectivity for supplementary motor area (SMA), pre-supplementary motor area (pre-SMA) and dorsolateral prefrontal cortex (dlPFC) are plotted for 5 points along an anterior – posterior axis of the right putamen.

We assessed intrinsic CBGTC organization (without covariates) by examining connectivity between carefully defined prefrontal functional regions and striatum and secondly, striatum with basal ganglia subregions and thalamus in the same healthy volunteers. The fronto-striatal map demonstrates that functionally defined prefrontal cortical regions have connectivity with dissociable sub-regions of the striatum (Fig. 1, Fig. 2, Fig. 3). We show predominant connectivity of ventral and mesial prefrontal cortical regions (medial and lateral OFC, ventromedial PFC, sgACC, dorsal cingulate and dorsomedial PFC) with the VS; lateral prefrontal (dlPFC, IFC) with caudate; pre-SMA with anterior putamen; and SMA with posterior putamen. Statistics are reported in Table 1 and further details of whole brain corrected statistics are reported in Table S1. Gradient patterns of connectivity through the striatum were examined for all cortical regions (Fig. 2, Fig. 3). Parameter estimates of connectivity were extracted from 7 points along striatum from dorsal caudate (point 1) to VS (point 4) to dorsal putamen (point 7) (Fig. 2). While ventromedial and anterior cingulate cortical regions showed similar patterns of connectivity with peaks in VS, regions of heterogeneous function had varied patterns of gradiented connectivity. Connectivity of dlPFC, pre-SMA and SMA, as determined with one-way ANOVA, was significantly different for mid caudate [point 2, *F*_(2,188)_ = 7.660, *p* = .001], VS [point 4, *F*_(2,188)_ = 4.344, *p* = .014], and mid putamen [point 6, *F*_(2,188)_ = 4.001, *p* = .020]. Tukey post-hoc comparisons revealed that for mid caudate, connectivity for SMA was significantly lower than pre-SMA (*p* = .001) and dlPFC (*p* = .009); for VS, connectivity of dlPFC was lower than of pre-SMA (*p* = .029) and SMA (*p* = .027) and for mid putamen, connectivity of pre-SMA was significantly higher than dlPFC (*p* = .019). An anterior – posterior gradient of cortical connectivity within putamen was also examined for dlPFC, pre-SMA and SMA, which all showed relatively high putaminal connectivity (Fig. 2). As expected, SMA had increasing connectivity estimates with more posterior regions of the putamen, whereas pre-SMA and dlPFC had the opposite pattern (Fig. 4). Connectivity of these cortical regions, determined with one-way ANOVA, was significantly different for anterior [point 1, *F*_(2,185)_ = 5.991, *p* = .003], and posterior putamen [point 5, *F*_(2,185)_ = 7.541, *p* = .001], but not mid putamen [point 3, *F*_(2,185)_ = 2.781, *p* = .065]. Post-hoc Tukey test demonstrated that for the anterior putamen, the pre-SMA had higher connectivity compared to both SMA (*p* = .003) and dlPFC (*p* = .049), and for posterior putamen, the SMA had higher connectivity compared to both pre-SMA (*p* = .013) and dlPFC (*p* = .001).

**Fig. 5:**
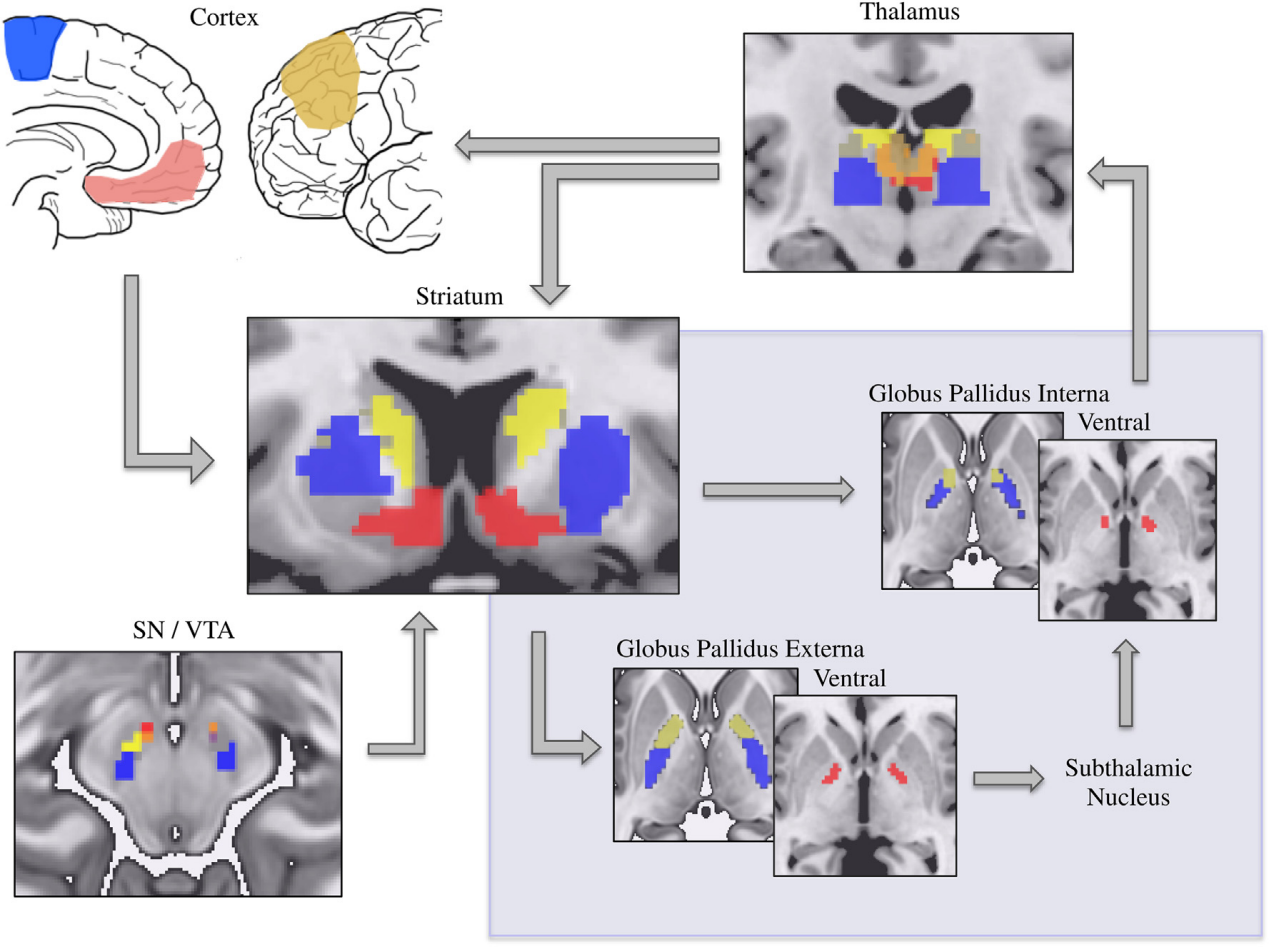
Cortico – basal ganglia – thalamic circuitry. Connectivity of the ventromedial prefrontal cortex (red), dorsolateral prefrontal cortex (yellow) and supplementary motor are (blue) with the ventral striatum, caudate and putamen, respectively is recapitulated. Seeds in ventral striatum, posterior putamen and dorsal caudate subsequently demonstrated distinct connectivity with globus pallidus interna and externa, substantia nigra/ventral tegmental area (SN/VTA) and thalamus. The blood-oxygen-level dependent (BOLD) overlays are shown using basal ganglia subregion and thalamus masks at family wise error corrected *p* < .005 for illustration purposes. See Supplementary Table 2 for statistics.

Fig. 5 reiterates the fronto-striatal connectivity for the ventromedial PFC (red), dlPFC (yellow) and SMA (blue) with the VS, caudate and putamen, respectively. We placed seeds in VS, posterior putamen and dorsal caudate to further examine dissociations of intrinsic connectivity within CBGTC circuitry focussing on the globus pallidus interna (GPi) and externa (GPe), thalamus and substantia nigra. Striatal seeds were dissociable in connectivity to GPe and GPi: ventral striatal connectivity was predominantly to ventral pallidum whereas posterior putamen was predominantly to the motor posterodorsal pallidum and dorsal caudate was predominantly functionally connected to anterodorsal pallidum (Fig. 5, Table S2). Also reflecting functionally relevant segregations, activity in both posterior putaminal and dorsal caudate seeds were correlated with bilateral lateral substantia nigra and the ventral striatal seed was correlated with right mesial midbrain compatible with the ventral tegmental area. Finally while the VS was functionally connected with the mediodorsal (MD) nucleus of the thalamus (associated with emotional, limbic processing), the posterior putamen showed connectivity with ventrolateral regions of the thalamus (associated with motor and somatomotor function). Connectivity between striatal subregions and subthalamic nucleus has been previously reported (L. S. Morris et al., 2015) and follows a similar dissociation of ventral striatal connectivity with mesial and putaminal connectivity with lateral subthalamic nucleus.

### 3.2 Study 2: behavioural functional correlates

**Fig. 6:**
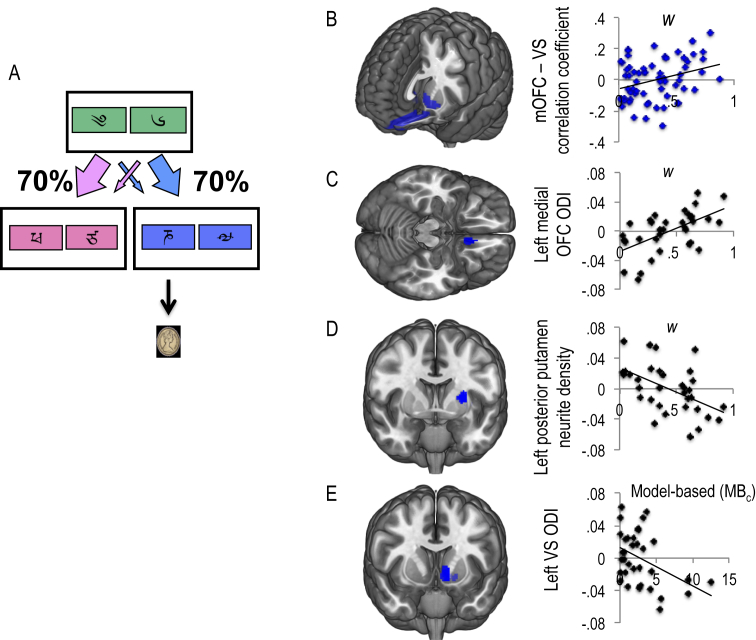
Neural correlates of *w*. A. Two step task: A choice between two stimuli at Stage 1 led with fixed probability (*p* = 70%) to one of two Stage 2 stimulus-pairs. A choice at Stage 2 probabilistically led to reward. B, *w*, the relative contribution of either model-based (*w* = 1) or model-free learning (*w* = 0) positively correlated with connectivity between ventral striatum (VS) medial orbitofrontal cortex (mOFC). C, *w* also positively correlated with mOFC orientation dispersion index (ODI) and D, negatively with left posterior putamen neurite density (adjusted parameter estimates plotted). A computational measure of model-based learning (MB_c_) negatively correlated with left VS ODI.

**Fig. 7:**
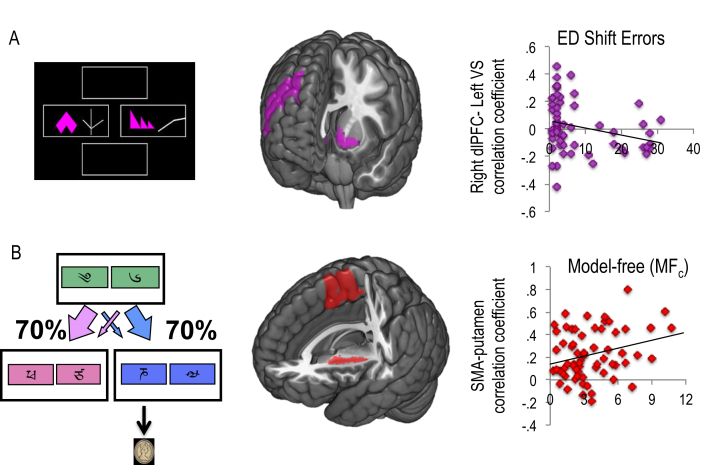
Latent substrates of attentional shifting and model-free learning. A, Extradimensional (ED) set-shifting requires attentional shifts to a previously irrelevant stimulus (i.e., shape *vs* line, left). Set shifting errors negatively correlated with connectivity between dorsolateral prefrontal cortex (dlPFC) and ventral striatum (VS). B, A computational measure of model-free learning (MF_c_) positively correlated with connectivity between supplementary motor area (SMA) and posterior putamen.

**Fig. 8:**
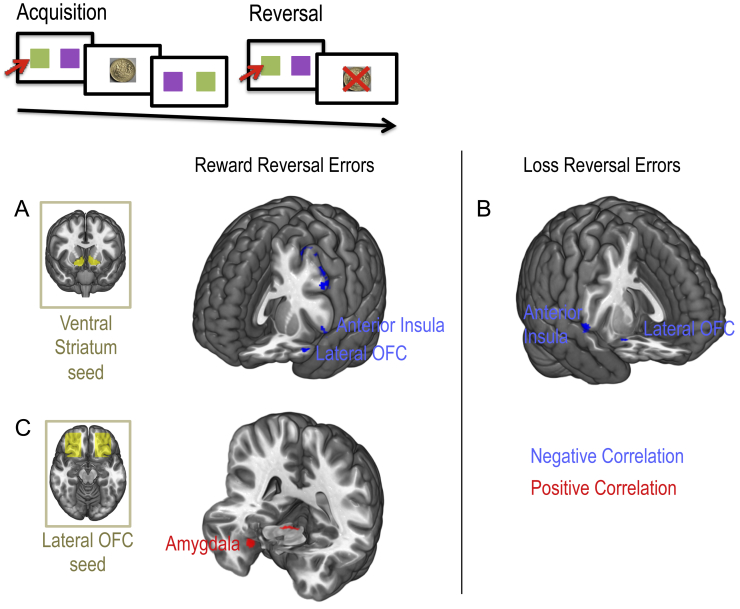
Neural correlates of reversal learning errors. Top: Reversal learning task. Ventral striatum seed-based functional connectivity maps revealed sites of connectivity that negatively correlated with both reward reversal errors (A) and loss reversal errors (B). C. Lateral orbitofrontal (OFC) seed-based connectivity correlated positively with reward reversal learning errors in the amygdala region. Cluster extent threshold correction was used for correlations with behaviour measures.

The following behavioural measures were examined in healthy volunteers: *w* (group mean, .31; standard deviation – SD, .23), ED shift errors [7.92; 9.44 SD), MBc (1.77; 1.76 SD), MF_c_ (3.55; 2.45 SD], reversal-learning trials to criterion for reward (345.65; 296.62 SD) and loss (3.45.51; 297.04 SD). We confirmed that the primary outcome measure, *w* or more goal-directed model-based behaviour was positively correlated with connectivity between medial OFC and VS (*R* = .32, *p* = .01; Fig. 6B). We also examined cognitive inflexibility in the form of ED set shifting errors. We did not find a significant correlation between ED errors and connectivity between dlPFC and caudate (*R* = .032, *p* = .811). However, ED shift errors were correlated with connectivity between dlPFC and VS (*R* = −.298, *p* = .021; Fig. 7A). In our exploratory analysis, we show that MF_c_ scores were positively correlated with connectivity between posterior putamen and SMA (*R* = .266, *p* = .033; Fig. 7B). Flexible updating of reward and loss stimulus-outcome contingencies was also examined using a probabilistic reversal learning task (Fig. 8, top). In the context of reward, the number of trials to criterion for reversal learning was negatively correlated with VS seed and lateral OFC and ventral anterior (VA)/mid insula (Fig. 8A) and positively correlated with connectivity between the lateral OFC seed and the amygdala (Fig. 8C). In the context of loss, slower reversal-learning also negatively correlated with VS and lateral OFC and dorsal anterior/mid insula connectivity (Fig. 8B) (see Table 2 for statistics).

### 3.3 Study 3: neurite orientation dispersion and density

We also show that *w* was negatively correlated with neurite density in the left posterior putamen (SVC FWE peak coordinates, −28, 0, 14; *Z* = 3.29; *p* = .036) and right SMA (peak coordinates, 6, −2, 48; *Z* = 3.69; *p* = .032) (Fig. 6C, D). Mb_c_ scores correlated negatively with left VS orientation dispersion (SVC FWE, peak coordinates, −8, 12, −4; *Z* = 3.73; *p* = .006) (Fig. 6E).

## 4 Discussion

We developed CBGTC maps based on functionally defined frontal cortical regional connectivity with striatum and functionally distinct striatal regions with basal ganglia subregions and thalamus. Previous resting-state connectivity studies have analysed cortico-striatal coupling focussing on 6 striatal seeds (Di Martino et al., 2008), striatal mapping using a clustering algorithm involving a 17 network parcellation of the entire cerebral cortex (Choi et al., 2012), and striatal parcellation using a clustering algorithm involving the entire cerebral cortex (Jung et al., 2014). Here we expand on this literature, using a novel multi-echo acquisition and analysis (Kundu et al., 2012) focussing on carefully defined frontal seed regions based on function and elucidating the pathway of subcortical connectivity couplings. We show dissociable intrinsic fronto-striatal connectivity with predominant connectivity between ventral and medial prefrontal regions with VS, lateral prefrontal regions with caudate, pre-SMA with anterior putamen and SMA with posterior putamen. We further demonstrate opposing gradient patterns of connectivity for SMA and pre-SMA along an anterior – posterior axis of the putamen. Regions implicated in multiple functions have connectivity across multiple striatal subregions (e.g., pre-SMA and IFC has connectivity to all striatal subregions; subgenual and dorsal cingulate has connectivity to all subregions except posterior putamen) but limbic ventromedial and anterior cingulate regions maintain clear preference of connectivity with VS over caudate and putamen. Similarly downstream, limbic, cognitive and motor connectivity respected a mesial-lateral division of the substantia nigra/ventral tegmental area and ventral-dorsal and anterior–posterior division of the globus pallidus: motor and cognitive striatal regions connected to dorsal pallidum and limbic regions to ventral pallidum. In the thalamus, the VS was functionally connected with the MD nucleus, which, along with additional inputs from ventral pallidum and amygdala, mediates limbic processes. On the other hand, the posterior putamen connected with the ventral lateral (VL) and VA nuclei, which, via connections with cerebellum and cortical motor areas, are involved with motor feedback and planning, respectively (Alexander and Crutcher, 1990, Behrens et al., 2003).

We emphasize a functional role of these fronto-striatal connections by showing that prospective model-based goal-directed learning is associated with the latent biomarker of medial OFC and ventral striatal intrinsic connectivity during rest as well as enhanced medial OFC neurite complexity. Neurite density and complexity is associated with the hierarchy of computations performed by neural structures (Jacobs et al., 2001). For example, heteromodal regions recruited for later stages of information processing consist of more complex dendrite and spine features than primary and unimodal cortical regions (Jacobs et al., 2001). Diffusion imaging and modelling in vivo is consistent with both Golgi staining of individual axonal and dendritic processes (Jespersen et al., 2012) and microscopic detailing of grey matter neurite density and dendritic architecture (Jespersen et al., 2010), highlighting the coherence between gross brain imaging and microscopic cellular profiling.

These findings suggest a neural network for MB_c_ involving integration of instrumental performance (VS) and flexible, computationally-driven updating of outcome value based on changing internal motivational states and external feedback (medial OFC). The results dovetail with recent findings using structural and on-task functional neuroimaging, including a report of correlations between MB_c_ and higher medial OFC volume (Daw et al., 2011, Glascher et al., 2010, Lucantonio et al., 2012, Smittenaar et al., 2013, Voon et al., 2014).

On an exploratory basis, we found a dissociation between model-based and retrospective model-free habit learning implicating putaminal neurite density and functional connectivity between the putamen and SMA. The dissociability of the networks underlying model-based and MF_c_ supports the intrinsic parcellation of this fronto-striatal map. Most previous findings from studies of computational learning behaviour have focused on the neural correlates of MB_c_ or higher *w* scores (Smittenaar et al., 2013), with fewer clear correlates of MF_c_. Consistent with the hypothesis that MF_c_ gives rise to habits, the current findings emphasize the similarities in the neural correlates underlying MF_c_ as operationalized computationally, with that for conventional habit learning in rodent lesion studies (Yin et al., 2004) and human imaging studies (Tricomi et al., 2009, de Wit et al., 2012) based on overtraining and testing of sensitivity to devaluation. Our results in this respect also converge with a recent report of on-task functional neuroimaging using a three-step decision tree task, related to the current task in which model-free values were shown to be encoded by the putamen (Wunderlich et al., 2012).

ED shift errors, a conceptual attentional shift, were associated with reduced functional connectivity between dlPFC and VS. The role of the dlPFC in ED shifting is well-established implicating a capacity for storing multiple choice options during outcome evaluation (Dias et al., 1996, Haber and Knutson, 2010, Manes et al., 2002). Our present finding converges with non-human primate studies showing only a limited role of the caudate nucleus in ED shifting (Collins, Wilkinson, Everitt, Robbins, & Roberts, 2000) and rodent studies showing that nucleus accumbens lesions can impair strategy shifting (Block, Dhanji, Thompson-Tardif, & Floresco, 2007). Specifically, nucleus accumbens core lesions can impair the later stage of acquisition and maintenance of a new strategy rather than the capacity to shift away from previously learned contingencies (Floresco, Ghods-Sharifi, Vexelman, & Magyar, 2006). Thus, our findings may represent this later stage of acquisition and maintenance of a new strategy. We further show that slower reversal learning across both reward and loss valences is negatively correlated with lateral OFC and VS connectivity. Several lines of evidence implicate the lateral OFC specifically in reversal learning. Depletion of serotonin in primate OFC is associated with reversal impairments (Groman et al., 2013) and deep brain stimulation of the lateral OFC in rodents impairs spatial reversal learning but not acquisition learning (Klanker, Post, Joosten, Feenstra, & Denys, 2013). In human studies, activation of the lateral OFC is specifically involved with reversal implementation (Hampshire et al., 2012).

Together, we show the parcellation of discrete functional regions of CBGTC and in particular, fronto-striatal circuitry and highlight dissociable intrinsic networks underlying goal-directed and habitual behaviour in healthy volunteers. We further provide evidence of anatomical segregation of functional regions of this circuitry with connectivity of functionally defined prefrontal cortical regions projecting to dissociable motor, limbic and associative striatal regions (Middleton and Strick, 2000, Parent and Hazrati, 1995).

## Figures and Tables

**Table 1:** Prefrontal intrinsic resting-state connectivity with striatal subregions. Reported as small volume family wise error corrected. Abbreviations: dlPFC, dorsolateral prefrontal cortex; vmPFC, ventromedial prefrontal cortex; SMA, supplementary motor area; pre-SMA, pre-supplementary motor area; mOFC, medial orbitofrontal cortex; lOFC, lateral orbitofrontal cortex; antrPFC, anterior prefrontal cortex; dmPFC, dorsomedial prefrontal cortex; IFC, inferior frontal cortex; sgACC, subgenual anterior cingulate cortex; dACC, dorsal anterior cingulate cortex; SVC p(FWE-corr), small volume corrected (*p* < .05) family-wise error *p* value; Z, Z-score; xyz, peak voxel coordinates.

Seed	ROI	SVC p(FWE-corr)	Z	x	y	z
dlPFC	Dorsal caudate	.001	4.47	15	12	11
Ventral striatum	.005	4.16	−13	17	0
Anterior putamen	ns				
Posterior putamen	ns				
vmPFC	Ventral striatum	<.001	6.64	−6	12	−10
Anterior putamen	.006	4.05	−13	5	−12
Dorsal caudate	ns				
Posterior putamen	ns				
SMA	Posterior putamen	<.001	>8.0	−38	3	7
Anterior putamen	<.001	7.01	27	0	9
Ventral striatum	.019	3.8	−22	10	−3
Dorsal caudate	ns				
pre-SMA	Anterior putamen	<.001	7.54	17	12	4
Posterior putamen	<.001	6.79	−34	7	7
Ventral striatum	<.001	6.72	−13	10	0
Dorsal caudate	<.001	6.26	15	10	11
mOFC	Ventral striatum	.001	4.55	6	12	−17
Anterior putamen	ns				
Posterior putamen	ns				
Dorsal caudate	ns				
lOFC	Ventral striatum	<.001	7.52	15	14	−17
Dorsal caudate	<.001	5.37	−27	12	−14
Anterior putamen	ns				
Posterior putamen	ns				
antrPFC	Ventral striatum	<.001	5.04	−10	17	0
Dorsal caudate	<.001	4.17	13	14	9
Anterior putamen	ns				
Posterior putamen	ns				
dmPFC	Ventral striatum	.001	4.66	−24	17	−14
Dorsal caudate	.045	3.86	−13	14	9
Anterior putamen	ns				
Posterior putamen	ns				
IFC	Dorsal caudate	<.001	6.95	−27	21	0
Posterior putamen	<.001	6.06	−38	0	2
Ventral striatum	<.001	5.59	−10	10	2
Anterior putamen	<.001	5.17	15	7	4
sgACC	Ventral striatum	<.001	>8.0	3	21	−5
Anterior putamen	<.001	7.42	−13	7	−12
Dorsal caudate	.005	4	−10	21	9
Posterior putamen	ns				
dACC	Ventral striatum	<.001	>8.0	8	24	−7
Anterior putamen	<.001	>8.0	−13	7	−12
Dorsal caudate	.009	3.82	13	14	18
Posterior putamen	ns				

**Table 2:** Neural connectivity correlates of learning errors for reward and loss. Whole brain connectivity maps for seed regions of interest (ROI) were correlated with reversal errors for reward and loss separately. Abbreviations: PFC, prefrontal cortex; OFC, orbitofrontal cortex; dlPFC, dorsolateral prefrontal cortex; Z, Z-score statistic following cluster extent thresholding.

Seed ROI	Correlation		Cluster	Z	x	y	z
*Reward reversal errors*							
Lateral OFC	Positive	Midbrain	25	4.13	6	−27	−12
Amygdala (right)	27	4.04	20	−2	−19
Negative	Frontal Polar	16	4.04	15	63	−12
Ventral striatum	Positive	Midbrain	18	4.01	−8	−27	−45
Negative	Parietal	198	5.47	−50	−69	−24
Cerebellum	26	3.94	24	−81	−45
Insula	24	3.75	−55	17	−5
Lateral PFC	132	3.73	−52	12	39
Lateral OFC	23	3.5	−43	31	−14
*Loss reversal errors*							
Lateral OFC	Positive	nil					
Negative	dlPFC	23	3.45	24	45	28
Ventral striatum	Positive	nil					
Negative	Insula	21	3.79	62	5	4
Temporal	18	3.75	−20	−11	−47
Lateral OFC	21	3.7	22	10	−19
