# Supplementary material for: Fronto-striatal organization: Defining functional and microstructural substrates of behavioural flexibility
Source: Cortex. 2016 Jan;74:118–33. doi: 10.1016/j.cortex.2015.11.004 (PMC4729321; doi:10.1016/j.cortex.2015.11.004)
Supplement: Supplementary file 1 [file mmc1.docx]

Supplementary materials

*Task descriptions*

The Intradimensional-extradimensional shift task (IDED shift; CANTAB(Downes et al., 1989); Figure 3) involves two stimulus feature dimensions of colour-filled shapes and white lines. Simple stimuli contain just one dimension, whereas compound stimuli contain both. There are a series of stages and six successive correct trials are necessary for continuation of each stage. Stages 1-5 involve simple stimulus discrimination with the development of an attentional set to a single stimulus dimension (shapes). The complexity of the stimulus increases as irrelevant lines become compounded with the relevant shapes. Stages 6 and 7 involve the introduction of new shapes, or intra-dimensional shift and reversal, respectively. The attentional set to a single salient stimulus dimension remains. At the crucial stage 8, the previously irrelevant stimulus dimension (lines) becomes relevant and subjects must perform an extra-dimensional (ED) shift by responding to the correct line and ignore the shape. Finally, stage 9 is an ED reversal. Thus, an ED shift requires shifting of attention from a previously relevant to a previously irrelevant dimension, requiring cognitive flexibility.

For the probabilistic reversal learning task, the acquisition phase included the following probabilistic outcomes: Loss (Stimulus A: P=0.30 Win +£1 / P=0.70 Lose -£2 (mean -£1.1); Stimulus B: P=0.70 Win +£1 / P=0.30 Lose -£2 (mean +£0.1)), Neutral (Stimulus C: P=0.70 Win +£1 / P=0.30 Lose -£1 (mean+£0.4); Stimulus D: P=0.30 Win +£1 / P=0.70 Lose -£1 (mean -£0.4)) or Reward (Stimulus E: P=0.70 Win +£2 / P=0.30 Lose -£1 (mean+£1,1); Stimulus F: P=0.30 Win +£2 / P=0.70 Lose -£1 (mean+0.1)). After 30 trials of each condition for acquisition, the contingencies were switched for the reversal phase (e.g. Stimulus A: P=0.70 Win +£1 / P=0.3 Lose -£2; Stimulus B: P=0.30 Win +£1 / P=0.70 Lose -£2). There were a total of 180 trials. The position of the stimuli within each stimulus-pair was counterbalanced on either side of the screen and the stimuli conditions were randomly presented. Subjects were given 10 practice trials of a stimuli-pair in which one stimulus was associated with P=0.70 Win +£1 / P=0.30 Lose -£1 and the other stimulus associated with P=0.30 Win +£1 / P=0.70 Lose -£1). The stimulus was 2.5 seconds during which the subjects responded with either the left arrow on the keyboard for the stimulus on the left or the right arrow on the keyboard for the stimulus on the right. If subjects were too slow, this was followed by the words: “You were too slow. Respond faster”.

*Computational modeling*

The following is adapted from Daw et al(Daw, Gershman, Seymour, Dayan, & Dolan, 2011). During the model-based model-free task, there are three states (stage-one state A (*s_A_*); stage-two state B and C (*s_B_* and *s_C_*)), each with two actions (*a_A_* and *a_B_*). In order to model the habitual learning strategy, we used a SARSA (λ) temporal difference (TD) algorithm where each choice is based on a predicted long-run value (Q*_TD_* (*s,a*)) for each action *a* at each stage *s*. In this framework, the TD reward prediction error (δ) informs subsequent predictions. For each trial *t* the stage-one state s_1,_*_t_* (s_A_) requires an action a_1,_*_t_* choice. The stage-two state s_2,_*_t_* (s_B_ or s_C_) also requires an action a_2,_*_t_* choice, leading to a reward *r*_2,_*_t_* (£1 or £0). After each stage *i* (1,2) of each trial *t* when the stage-two state is displayed and at final reward presentation, a prediction error δ*_i,t_* will occur. These will update the previous states s*_i,t_* value Q*_TD_* and action a*_i,t_*:

Q*_TD_* (*s_i.t_,a_i,t_*) = Q*_TD_* (*s_i.t_,a_i,t_*) + α*_i_*δ*_i,t_*

where

δ*_i,t_* = *r_i,t_* + Q*_TD_* (*s_i_*_+1_*_,t_*,*a_i_*_+1_*_,t_*) - Q*_TD_* (*s_i_*_,_*_t_*.*a_i,t_*)

This updates the action value of stage-one depending on the value following the stage-two state, Q*_TD_* (*s*_2_*_,t_,a*_2,_*_t_*). *r*_1,_*_t_*=0 because no reward is received at this stage. The reward r_2,_*_t_* then updates the value at the second stage. Here, the terminal value Q*_TD_* (*s*_3_*_,t_,a*_3,_*_t_*)=0. A separate parameter is used for the learning rate for the update of each stage (α_1_, α_2_). The stage-one action value is updated by the stage-one prediction error described but also according to the stage-two prediction error at the end of each trial when the reward *r*_2,_*_t_* is received, which is added to the previous:

Q*_TD_* (*s*_1,_*_t_*, *a*_1,_*_t_*) = Q*_TD_* (*s*_1,_*_t_*, *a*_1,_*_t_*) + α_1_λδ_2,_*_t_*

This update extent is also determined by the eligibility trace parameter λ.

At stage-one (*Q_MB_*), the model-based reinforcement learning algorithm calculated the action value per action based on the probabilities that the current action would lead to each stage two state (*P*(*s_B_*|*s_A_.a_A_*)=0.70; (*P*(*s_B_*|*s_A_.a_A_*)=0.30; and conversely for *s_C_*) and the values of those states. Therefore, for each action *a_j_* (*j*= *A,B*):

*Q_MB_* (s*_A_*,a*_j_*) = *P*(*s_B_*|*s_A_,a_j_*)max_k_*Q_TD_*(*s_B_,a_k_*) + *P*(*s_C_*|*s_A_,a_j_*)max_k_*Q_T_*_D_(*s_C_,a_k_*)

The stage-two value here is equivalent to the model-free value of the optimal action as both model-free and model-based values coincide at the end state. For each stage-one action, a net action value is calculated depending on the weighted sum of both model-free and model-based values:

*Q_net_*(*s_A_,a_j_*) = *wQ_MB_*(*s_A_,a_j_*) + (1 – *w*)*Q_TD_*(*s_A_,a_j_*)

where *w* is a weighting parameter such that higher *w* (*w*=1) indicates reliance on model-based learning strategies while lower *w* (*w*=0) indicates greater reliance on model-free. At stage two, *Q_NET_*=*Q_TD_*. For each stage, the probability of a choice is calculated using the softmax equation in *Q_net_*:

*P*(*a_i,t_* = *a*|*s_i,t_*) α exp ( *β_i_*[*Q_net_*(*s_i_*_,_*_t_,a*) + *p* * *rep*(*a*)] )

where *β_i_* is an index of choice reliability at each stage (β_1,_ β_2_) with higher values indicating higher reliability. *p* accounts for perseveration (*p*>0) or switching (*p*<0) of choices in stage one. *rep*(*a*) acts as a binary indicator such that it has a value of 1 if *a* is an action from stage one and *a* = *a*_1_,*_t_*_-1_, and otherwise equals 0. We estimated the free parameters of the model (*w*, *p,* α_1_, α_2_, β_1,_ β_2_, p, λ) for each subject separately by maximum likelihood (over the joint probability of each choice conditional on the preceding choice and outcomes) as described in Daw et al. (2011). Complementing *w*, which characterizes the net contribution of model-based vs. model-free values to choice, we computed the scores MB=w β_1,_ and MF=(1-w) β_1,_, as the unscaled contribution of each value separately to choice, producing model-based computation (MB_c_) and model-free computation (MF_c_) scores. Accordingly, the relativized score *w* = MB/(MF + MB).

In the computational model, parameter values can be determined by integrating the effects associated with choice sequences. One way of determining behaviour is to examine pairs of successive choices, for example, the tendency to Stay with or Switch from a Stage 1 choice after an Outcome (Reward or No Reward) which depends on the Frequency of the Stage 1 to Stage 2 transition (Common (P=0.70) or Rare (P=0.30)). Habitual behaviour would be defined as a repeated Stage 1 choice (Stay) after a Reward, regardless of a Common or Rare transition. Goal-directed behaviour would more likely entail a Switch from a Stage 1 choice if it was Rewarded but the transition was Rare. With a knowledge of the task structure, the alternate Stage 1 choice would lead to a Reward following a Stage 2 choice with higher likelihood. A habitual strategy would reflect a main effect of Outcome in Stay probability whereas a goal-directed strategy would reflect an interaction between Outcome x Frequency. Thus, model-based behaviour (MB_b_) is a measure of the strength of the interaction in the subject and model-free behaviour (MF_b_) is a measure of the main effect of Outcome.

*Imaging analysis*

For the intrinsic connectivity of prefrontal and striatal regions, we computed the individual beta maps for carefully defined prefrontal ROI regions as ROI-to-voxel analyses. To calculate statistics, we assessed the beta maps at the group level focusing only on striatal connectivity. We report both the whole brain family wise error (FWE) P<0.05 corrected striatal peak voxel statistics (Table S1) and small volume corrected (SVC) FWE P<0.05 corrected statistics for different regions of the striatum (ventral striatum, caudate, anterior putamen and posterior putamen) (Table S2).

*Seed definition*

The bilateral ventral striatal (VS) anatomical ROI, previously used in other studies(Murray et al., 2008) had been hand drawn using MRIcro based on the definition of VS(Martinez et al., 2003). The putamen ROI was obtained from the Automated Anatomical Labelling (AAL) atlas. The posterior putamen was separated from anterior putamen using a vertical line passing through the anterior commissure. The caudate ROI was obtained from the AAL atlas with removal of the ventral striatal ROI.

The following seeds were manually created or altered using MarsBaR ROI toolbox(Brett M, 2002) for SPM(Brett M, 2002). For the orbitofrontal cortex (OFC), the dorsal extent was defined by the axial slice showing the disappearance of the olfactory sulcus, the medial and lateral OFC were distinguished by the crown of the gyrus rectus(Cox et al., 2014). The lateral OFC consisted of two boxes of 14.2 x 22 x 2mm centered on coordinates ±28, 36, -18. The medial OFC (mOFC) ROI consisted of the combination of 2 boxes, the size of 6 x 26 x 4mm and centered on coordinates ±6, 36, -22. The vmPFC was defined anteriorly by the posterior border of the cytoarchitectonic anterior prefrontal cortex (i.e., the most anterior coronal slice in which all three frontal gyri are visible), also known as area 10p (Ongur, Ferry, & Price, 2003); posteriorly and laterally by the cingulate cortex; dorsally by the genu of the corpus callosum; and ventrally by the superior boundary of the mOFC described above. To make the dlPFC ROI, we combined the masks of Brodmann areas 46 and 9 from the AAL atlas, and manually restricted the mask to the boundaries of the dlPFC. The anterior border was defined by the most anterior tip of the corpus callosum (CC); the posterior extent was the posterior border of the genu of the CC; the ventral border was the inferior frontal sulcus; and the medial border defined by the cingulate sulcus(Cox et al., 2014; Sanches et al., 2009). To create the SMA seed, we modified the SMA ROI from the AAL atlas. The posterior border of the pre-SMA is typically defined as a vertical line through the anterior commissure, the anterior border defined as a vertical line passing through the genu of the corpus callosum and the inferior border being the superior border of the cingulate cortex (Kim et al., 2010). The posterior border of the SMA is defined by the primary motor cortex. By respecting these boundaries, we created the SMA ROI.

We used Brodmann Area 25 from AAL atlas to create the subgenual cingulate seed. For the dorsal anterior cingulate cortex, the cingulate cortex ROI from AAL atlas was modified such that the anterior border was defined as the tip of the genu of the corpus callosum (Cox et al., 2014; Desikan et al., 2006) and the posterior was the posterior end of the genu of the corpus callosum (Desikan et al., 2006). The inferior frontal cortex (IFC) ROI was created using the inferior frontal sulcus as the superior boundary; the precentral gyrus as the posterior boundary(Cox et al., 2014; Desikan et al., 2006); and the rostral extent of the inferior frontal sulcus as the anterior boundary(Desikan et al., 2006). This was then restricted to those regions falling within a 300mm radius sphere on x= ±48, y=18, z=8 (Johnson-Frey et al., 2003), using the anterior horizontal ramus of the Sylvian fissure to differentiate from orbital regions(Cox et al., 2014). For the anterior prefrontal cortex (PFC) ROI we used Brodmann area 10 from WFU PickAtlas and manually restricted the ROI posteriorly at the boundary of the anterior coronal place where the three frontal gyri are present(Ongur et al., 2003; Ongur & Price, 2000; Ramnani & Owen, 2004), and dorsally by the dorsal extent of area 10p described by(Ongur et al., 2003). The dorsomedial prefrontal cortex ROI was created using the dorsal boundary of the anterior PFC, the lateral boundaries described for the vmPFC and anterior boundary described for the pre-SMA.

Exploratory analyses of gradient patterns of cortical connectivity through the striatum were performed for dlPFC, pre-SMA and SMA. First for illustration, parameter estimates of connectivity for each frontal cortical seed with the striatum were computed at 7 points along coronal slice 12 (Figure 1) of the right striatum: Point 1, xyz= 15, 12, 16; point 2, xyz=11, 12, 8; point 3, xyz=9, 12, 0; point 4, xyz=11, 12, -10; point 5, xyz=20, 12, -7; point 6, xyz= 23, 12, -2; point 7, xyz= 25, 12, 4. Between point 1 and 4, there were 8mm gaps in the z plane and between point 5 and 7, there were approximately 8mm gaps split between the x and z planes. Thus, points were roughly equidistant. Slice 12 was chosen from Figure 1 as it contained the most heterogeneity of cortical – striatal connectivity. A similar approach to examining graduated cortical connectivity was taken for the anterior – posterior axis of the right putamen, from anterior to posterior: point 1, xyz=26, 18, 0; point 2, xyz=25, 12, 0; point 3, xyz=28, 6, 0; point 4, xyz=31, 0, 0; point 5, xyz=32, -6, 0. The dlPFC, pre-SMA and SMA were chosen for these analyses as they demonstrate varied connectivity patterns with caudate and putamen that warranted further exploration and had functional relevance within the current study. For statistical analysis, striatal gradient points 2, 4 and 6 and putamen gradient points 1, 3 and 5 were chosen as equidistant points that reduced the likelihood of connectivity overlaps.

Table 1. Prefrontal intrinsic resting state connectivity with striatum (whole brain statistics).

| Seed | Striatal Peak Regions | Whole brain  p(FWE-corr) | Z | x | y | z |
| --- | --- | --- | --- | --- | --- | --- |
| dlPFC | Caudate | <0.001 | 5.61 | 31 | 19 | 0 |
| vmPFC | Ventral striatum, cluster includes anterior cingulate cortex | <0.001 | >8.0 | -1 | 49 | -7 |
| SMA | Putamen, cluster includes pre-SMA, motor cortex, insula | <0.001 | >8.0 | 3 | -11 | 65 |
| preSMA | Putamen, cluster includes caudate and ventral striatum | <0.001 | >8.0 | -41 | 14 | 0 |
| antrPFC | Caudate | 0.024 | 4.97 | 13 | 17 | 4 |
| IFC | Caudate | 0.001 | 5.66 | -13 | 10 | 4 |
| sgACC | Ventral striatum, cluster extends to vmPFC, caudate and amygdala | <0.001 | >8.0 | -3 | 17 | -7 |
| dACC | Ventral striatum, cluster includes caudate, vmPFC | <0.001 | >8.0 | -1 | 35 | -5 |

Table 1. Prefrontal intrinsic resting state connectivity with striatum. Abbreviations: dlPFC, dorsolateral prefrontal cortex; vmPFC, ventromedial prefrontal cortex; SMA, supplementary motor area; preSMA, pre- supplementary motor area; antrPFC, anterior prefrontal cortex; IFC, inferior frontal cortex; sgACC, subgenual anterior cingulate cortex; dACC, dorsal anterior cingulate cortex; p(FWE-corr), whole brain (P<0.05) family-wise error P value; Z, Z-score; xyz, peak voxel coordinates.


Table S2. Striatal connectivity with basal ganglia subregions and thalamus.

| Seed | ROI | SVC p(FWE-corr) | Z | x | y | z |
| --- | --- | --- | --- | --- | --- | --- |
| Posterior Putamen | Thalamus | <0.001 | >8 | 22 | -20 | 11 |
|  | Gpe | <0.001 | >8 | 24 | -7 | 2 |
|  | Gpi | <0.001 | 7.31 | -13 | 0 | 2 |
|  | SN / VTA  STN* | <0.001  <0.001 | 5.79  6.29 | 15  13 | -18  -16 | -7  -5 |
| Ventral Striatum | Thalamus | <0.001 | 7.72 | -3 | -9 | 7 |
|  | Gpe | <0.001 | >8 | 13 | 7 | -5 |
|  | Gpi | <0.001 | >8 | -10 | 3 | -5 |
|  | SN / VTA  STN* | 0.049  0.035 | 2.84  2.72 | 6  8 | -11  -11 | -14  -5 |
| Dorsal Caudate | Thalamus | <0.001 | >8 | 15 | -9 | 16 |
|  | Gpe | <0.001 | >8 | -13 | 3 | 7 |
|  | Gpi | <0.001 | >8 | -10 | 3 | 2 |
|  | SN / VTA  STN | 0.047  ns | 2.48 | 13 | -20 | -12 |

Table S2. Striatal connectivity with basal ganglia subregions and thalamus. Reported as small volume family wise error corrected. Abbreviations: GPe, globus pallidum externa; GPi, globus pallidum interna; SN / VTA, substania nigra / ventral tegmental area; STN, subthalamic nucleus; * previously reported; ns, not significant; SVC p(FWE-corr), small volume corrected (P<0.05) family-wise error P value; Z, Z-score; xyz, peak voxel coordinates.

References

Brett M, A. J., Valabregue R, Poline JB. (2002). Region of interest analysis using an SPM toolbox [abstract]. *Presented at the 8th International Conferance on Functional Mapping of the Human Brain, Available on CD-ROM in NeuroImage, Vol 16, No 2, abstract 497*.

Cox, S. R., Ferguson, K. J., Royle, N. A., Shenkin, S. D., MacPherson, S. E., MacLullich, A. M., . . . Wardlaw, J. M. (2014). A systematic review of brain frontal lobe parcellation techniques in magnetic resonance imaging. *Brain Struct Funct, 219*(1), 1-22. doi: 10.1007/s00429-013-0527-5

Daw, N. D., Gershman, S. J., Seymour, B., Dayan, P., & Dolan, R. J. (2011). Model-based influences on humans' choices and striatal prediction errors. *Neuron, 69*(6), 1204-1215. doi: 10.1016/j.neuron.2011.02.027

Desikan, R. S., Segonne, F., Fischl, B., Quinn, B. T., Dickerson, B. C., Blacker, D., . . . Killiany, R. J. (2006). An automated labeling system for subdividing the human cerebral cortex on MRI scans into gyral based regions of interest. *Neuroimage, 31*(3), 968-980. doi: 10.1016/j.neuroimage.2006.01.021

Downes, J. J., Roberts, A. C., Sahakian, B. J., Evenden, J. L., Morris, R. G., & Robbins, T. W. (1989). Impaired extra-dimensional shift performance in medicated and unmedicated Parkinson's disease: evidence for a specific attentional dysfunction. [Research Support, Non-U.S. Gov't]. *Neuropsychologia, 27*(11-12), 1329-1343.

Johnson-Frey, S. H., Maloof, F. R., Newman-Norlund, R., Farrer, C., Inati, S., & Grafton, S. T. (2003). Actions or hand-object interactions? Human inferior frontal cortex and action observation. *Neuron, 39*(6), 1053-1058. doi: Doi 10.1016/S0896-6273(03)00524-5

Kim, J. H., Lee, J. M., Jo, H. J., Kim, S. H., Lee, J. H., Kim, S. T., . . . Saad, Z. S. (2010). Defining functional SMA and pre-SMA subregions in human MFC using resting state fMRI: functional connectivity-based parcellation method. *Neuroimage, 49*(3), 2375-2386. doi: 10.1016/j.neuroimage.2009.10.016

Martinez, D., Slifstein, M., Broft, A., Mawlawi, O., Hwang, D. R., Huang, Y., . . . Laruelle, M. (2003). Imaging human mesolimbic dopamine transmission with positron emission tomography. Part II: amphetamine-induced dopamine release in the functional subdivisions of the striatum. [Research Support, Non-U.S. Gov't

Research Support, U.S. Gov't, P.H.S.]. *J Cereb Blood Flow Metab, 23*(3), 285-300.

Murray, G. K., Corlett, P. R., Clark, L., Pessiglione, M., Blackwell, A. D., Honey, G., . . . Fletcher, P. C. (2008). Substantia nigra/ventral tegmental reward prediction error disruption in psychosis. [Research Support, Non-U.S. Gov't]. *Mol Psychiatry, 13*(3), 239, 267-276. doi: 10.1038/sj.mp.4002058

Ongur, D., Ferry, A. T., & Price, J. L. (2003). Architectonic subdivision of the human orbital and medial prefrontal cortex. *J Comp Neurol, 460*(3), 425-449. doi: 10.1002/cne.10609

Ongur, D., & Price, J. L. (2000). The organization of networks within the orbital and medial prefrontal cortex of rats, monkeys and humans. *Cereb Cortex, 10*(3), 206-219.

Ramnani, N., & Owen, A. M. (2004). Anterior prefrontal cortex: insights into function from anatomy and neuroimaging. *Nat Rev Neurosci, 5*(3), 184-194. doi: 10.1038/nrn1343

Sanches, M., Caetano, S., Nicoletti, M., Monkul, E. S., Chen, H. H., Hatch, J. P., . . . Soares, J. C. (2009). An MRI-based approach for the measurement of the dorsolateral prefrontal cortex in humans. *Psychiatry Res, 173*(2), 150-154. doi: 10.1016/j.pscychresns.2009.02.007
